# Mortality risk after COVID-19 vaccination: A self-controlled case series study

**DOI:** 10.1016/j.vaccine.2024.02.032

**Published:** 2024-02-22

**Authors:** Stanley Xu, Lina S. Sy, Vennis Hong, Paddy Farrington, Sungching C. Glenn, Denison S. Ryan, Abraelle M. Shirley, Bruno J. Lewin, Hung-Fu Tseng, Gabriela Vazquez-Benitez, Jason M. Glanz, Bruce Fireman, David L. McClure, Laura P. Hurley, Onchee Yu, Michael Wernecke, Ning Smith, Eric S. Weintraub, Lei Qian

**Affiliations:** a Research and Evaluation, Kaiser Permanente Southern California, Pasadena, CA, United States; b Department of Health Systems Science, Kaiser Permanente Bernard J. Tyson School of Medicine, Pasadena, CA, United States; c School of Mathematics and Statistics, The Open University, Milton Keynes, UK; d HealthPartners Institute, Minneapolis, MN, United States; e Institute for Health Research, Kaiser Permanente Colorado, Denver, CO, United States; f Department of Epidemiology, University of Colorado School of Public Health, Aurora, CO, United States; g Kaiser Permanente Vaccine Study Center, Kaiser Permanente Northern California, Oakland, CA, United States; h Marshfield Clinic Research Institute, Marshfield, WI, United States; i Denver Health, Denver, CO, United States; j Kaiser Permanente Washington Health Research Institute, Kaiser Permanente Washington, Seattle, WA, United States; k Acumen LLC, Burlingame, CA, United States; l Center for Health Research, Kaiser Permanente Northwest, Portland, OR, United States; m Immunization Safety Office, Centers for Disease Control and Prevention, Atlanta, GA, United States

**Keywords:** Self-controlled case series, COVID-19 vaccines, All-cause mortality, non-COVID-19 mortality, Cardiac-related mortality

## Abstract

**Background:**

Although previous studies found no-increased mortality risk after COVID-19 vaccination, residual confounding bias might have impacted the findings. Using a modified self-controlled case series (SCCS) design, we assessed the risk of non-COVID-19 mortality, all-cause mortality, and four cardiac-related death outcomes after primary series COVID-19 vaccination.

**Methods:**

We analyzed all deaths between December 14, 2020, and August 11, 2021, among individuals from eight Vaccine Safety Datalink sites. Demographic characteristics of deaths in recipients of COVID-19 vaccines and unvaccinated individuals were reported. We conducted SCCS analyses by vaccine type and death outcomes and reported relative incidences (RI). The observation period for death spanned from the dates of emergency use authorization to the end of the study period (August 11, 2021) without censoring the observation period upon death. We pre-specified a primary risk interval of 28-day and a secondary risk interval of 14-day after each vaccination dose. Adjusting for seasonality in mortality analyses is crucial because death rates vary over time. Deaths among unvaccinated individuals were included in SCCS analyses to account for seasonality by incorporating calendar month in the models.

**Results:**

For Pfizer-BioNTech (BNT162b2), RIs of non-COVID-19 mortality, all-cause mortality, and four cardiac-related death outcomes were below 1 and 95 % confidence intervals (CIs) excluded 1 across both doses and both risk intervals. For Moderna (mRNA-1273), RI point estimates of all outcomes were below 1, although the 95 % CIs of two RI estimates included 1: cardiac-related (RI = 0.78, 95 % CI, 0.58–1.04) and non-COVID-19 cardiac-related mortality (RI = 0.80, 95 % CI, 0.60–1.08) 14 days after the second dose in individuals without pre-existing cancer and heart disease. For Janssen (Ad26.COV2.S), RIs of four cardiac-related death outcomes ranged from 0.94 to 0.98 for the 14-day risk interval, and 0.68 to 0.72 for the 28-day risk interval and 95 % CIs included 1.

**Conclusion:**

Using a modified SCCS design and adjusting for temporal trends, no-increased risk was found for non-COVID-19 mortality, all-cause mortality, and four cardiac-related death outcomes among recipients of the three COVID-19 vaccines used in the US.

## 1 Background

Several cohort studies have consistently reported no-increased mortality risk after COVID-19 vaccination [1–4]. Moreover, two cohort studies carried out within the Vaccine Safety Datalink (VSD) demonstrated that COVID-19 vaccine recipients had lower non-COVID-19 mortality rates compared to unvaccinated individuals after adjusting for demographic characteristics in preliminary analyses [5] and individual- and community-level risk factors in subsequent analyses [6]. While no safety concern was identified from these analyses for non-COVID-19 mortality risk after COVID-19 vaccination, residual confounding bias likely remained after adjusting for several measured confounders. Vaccinated individuals may tend to be healthier and engage in fewer risky behaviors than unvaccinated comparators [7–9]. When assessing the association between vaccination and adverse outcomes, it is important to consider confounding factors influencing the relationship. These confounders can be both time-invariant and time-varying.

Compared to a cohort design with vaccinated versus unvaccinated individuals, the self-controlled case series (SCCS) design is considered to be less susceptible to the healthy vaccinee bias [10] due to its ability to control for time-invariant confounders. The original SCCS design was developed to study the association between transient exposures such as vaccination and acute outcomes such as febrile convulsions and aseptic meningitis [11]. In a SCCS study, the risk interval is a period after vaccination during which the risk for an adverse event may be increased while the observation time outside the risk interval is considered the control interval. Because incidence rates are compared within an individual, time-invariant covariates are adjusted for implicitly. Since its inception, the SCCS design has been widely employed in vaccine safety studies and other areas of research [12,13]. To properly evaluate adverse events including death that influence the likelihood of a subsequent exposure or the length of the observation period, a modified SCCS approach was developed within the counterfactual framework, using a pseudo-likelihood approach for estimation [14]. When death is the outcome, the modified SCCS approach employs planned end of observation as the actual end of observation for each case, rather than date of death.

Recently, two SCCS studies were conducted to investigate the association between COVID-19 vaccination and cardiac-related deaths. The first study was a non-peer-reviewed analysis conducted by the Florida Department of Public Health in 2022 using a SCCS design and posted on its website [15]. It reported that males aged 18–39 years had an increased risk of cardiac-related deaths in the 28 days following the last dose of mRNA COVID-19 vaccines (relative incidence [RI] = 1.97, 95 % confidence interval [Cl], 1.16–3.35). In contrast, a study by Nafilyan et al. in 2023 using the modified SCCS approach did not find an increased risk of cardiac-related deaths following mRNA COVID-19 vaccination among males aged 12–29 years in England after accounting for multi-dose administration of vaccine and adjusting for seasonality [16].

The goal of this study was to assess the mortality risk after a primary series of COVID-19 vaccination among individuals at eight VSD sites in the United States (US) using the modified SCCS design.

## 2. Methods

### 2.1. Study population and study period

For this SCCS study, analytic datasets were constructed using a cohort of individuals aged ≥ 12 years who were enrolled at eight VSD sites (Kaiser Permanente [KP] Southern California, KP Northern California, KP Colorado, KP Northwest, KP Washington, HealthPartners, Denver Health and Marshfield Clinic). To be included in the SCCS analyses, individuals had to have documented health plan enrollment on the emergency use authorization (EUA) date for their age group and had to have died between the corresponding EUA date and August 11, 2021. The EUA date for mRNA COVID-19 vaccines was December 14, 2020, for individuals aged ≥ 16 years, and May 10, 2021, for individuals aged 12–15 years. The EUA date for Janssen (Ad26.COV2.S) was February 27, 2021, for individuals aged ≥ 18 years. We chose August 11, 2021, as the end of the study because our focus was on mortality risk after a primary series of COVID-19 vaccination, and the EUA for the third mRNA dose (additional primary dose) among immunocompromised individuals was issued on August 12, 2021 [17].

### 2.2. Exposure

The exposure was documented receipt of the primary series COVID-19 vaccination: two doses of mRNA vaccines (Pfizer-BioNTech [BNT162b2] or Moderna [mRNA-1273]) or one dose of Ad26.COV2.S. Those who received only one dose of an mRNA vaccine and died were also included in the analyses. Vaccination status was assessed from December 14, 2020, for individuals aged ≥ 16 years and from May 10, 2021, for individuals aged 12–15 years through August 11, 2021, for mRNA vaccines, and from February 27, 2021, through August 11, 2021, for Ad26.COV2.S. The minimum recommended intervals between dose 1 and dose 2 were 17 days and 24 days (allowing for a 4-day grace period) for BNT162b2 and mRNA-1273, respectively. Any dose 2 administered less than 17 days and 24 days after dose 1, respectively, was considered invalid. Deaths of individuals who received different COVID-19 mRNA vaccines for doses 1 and 2 were excluded from the analyses.

### 2.3. Outcomes

The primary outcome was documentation of non-COVID-19-associated death during the study period. Non-COVID-19-associated deaths were defined as those that did not have an ICD-10 code U07.1 listed as a documented cause of death and did not occur within 30 days of a COVID-19 diagnosis, or a positive SARS-CoV-2 test. The study also had five secondary outcomes: all-cause death, cardiac-related death, cardiac-related death without pre-existing cancer and heart disease, non-COVID-19-associated cardiac-related death, and non-COVID-19-associated cardiac-related death without pre-existing cancer and heart disease (Supplementary Figure 1). Cardiac-related deaths were defined as those identified with the following ICD-10 codes for underlying cause of death: I00–I09, I11, I13, I20–I51 (Centers for Disease Control and Prevention/National Center for Health Statistics) [18]. Data from six out of the eight VSD sites were included in the analysis of cardiac-related death outcomes, as the remaining two did not have cause-of-death data available for the study period, although they had COVID-19 diagnosis codes and SARS-CoV-2 test data available for identifying non-COVID-19 deaths. For analysis of cardiac-related death outcomes without pre-existing cancer and heart disease, we required that individuals had ≥ 1-year health plan enrollment before the relevant EUA date for their age group to ensure potential capture of any history of cancer and heart disease within this timeframe.

### 2.4. Covariates

We collected demographic variables (age, sex, race/ethnicity) to describe the characteristics of the study population. Calendar time was included as a time-varying covariate.

### 2.5. Statistical analyses

We assessed mortality risk after vaccination for each of the three vaccines separately. Demographic characteristics of those individuals who died during the study period were described.

**Fig. 1.**
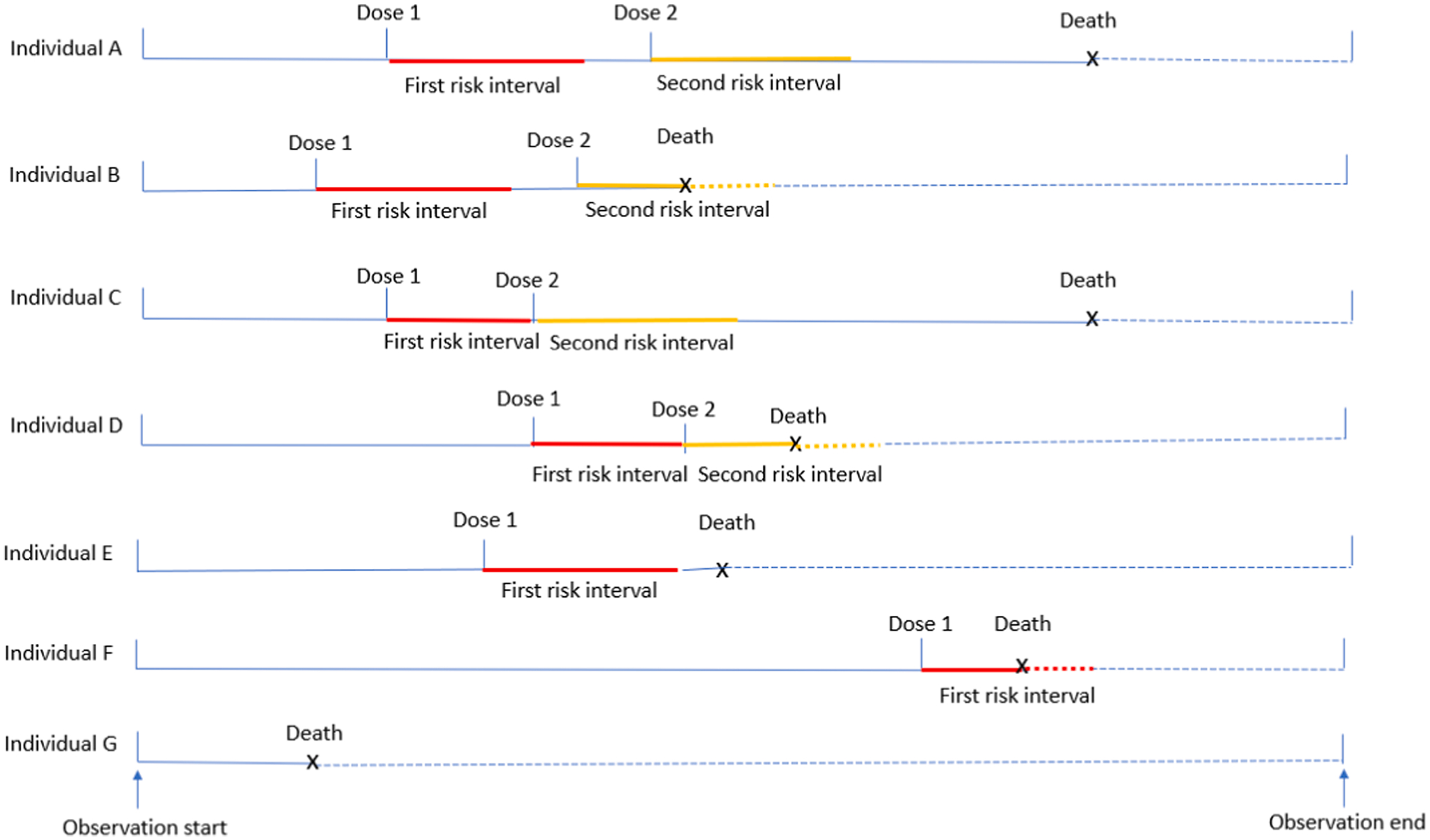
Scenarios for the timeline of administering one or two doses of a 2-dose primary series of mRNA COVID-19 vaccines, risk intervals and control intervals^$^ ^$^Individual A received two doses of mRNA vaccines and died during the control interval after dose 2; Individual B received two doses of mRNA vaccines and died during the second risk interval; Individual C received two doses of mRNA vaccines with dose 2 being administered before the end of the first risk interval, and died during the control interval after dose 2; Individual D received two doses of mRNA vaccines with dose 2 be administered before the end of the first risk interval, and died during the second risk interval; Individual E received only one dose of mRNA vaccine and died during the control interval after dose 1; Individual F received only one dose of mRNA vaccine and died during the first risk interval; Individual G was not vaccinated and died during the observation period. Dashed line represents person-time after death. In a modified self-controlled cases series design, follow-up continues after death.

Since death influenced both the observation period and subsequent potential vaccine exposure, we used a modified SCCS approach to analyze the primary and secondary outcomes. In the modified SCCS approach, the observation period for death was counted from the EUA date to the end of the planned study period (August 11, 2021), without censoring the observation period upon death. For the mRNA COVID-19 vaccines, there were two risk intervals, one for dose 1 and the other for dose 2. For Ad26.COV2.S, there was only one risk interval after dose 1. We pre-specified the primary risk interval to be 28 days (days 0 to 27) after each dose, and a secondary risk interval to be 14 days (days 0 to 13) after each dose. We also conducted additional analyses to estimate the overall and weekly relative incidences up to 10 weeks after each dose. Risk intervals started on the vaccination date because any deaths on day 0 must have occurred after vaccination by definition. The rest of the observation period (i.e., minus the risk interval) was the control interval. All conceivable scenarios for the timeline of administering one or two doses of a 2-dose primary series of mRNA COVID-19 vaccines, including risk intervals and control intervals, are illustrated in Fig. 1. If a second dose occurred during the 28-day risk interval for dose 1, the first risk interval was censored as demonstrated in Fig. 1 for Individuals C and D. To estimate the vaccination effect (RI), we employed a pseudo-likelihood approach implemented in R (Farrington et al, 2009) [14].

Deaths among unvaccinated individuals were included in the SCCS analyses to account for seasonality by including calendar month in the model [19]. Adjusting for seasonality in mortality analyses is crucial because death rates vary over time. The modified SCCS function in R can only accommodate one time-varying covariate. As age did not vary significantly in the relatively short observation period (less than 8 months) and SCCS adjusted for time-invariant covariates, age was not included in the model.

The modified SCCS function in R is not suitable for analyzing large numbers of all-cause and non-COVID-19 deaths. For all-cause and non-COVID-19 deaths after the primary series of mRNA vaccines, we randomly divided the sample into five subgroups. We obtained the vaccination effect coefficients from these five subgroup analyses and combined them using a fixed effect model in *meta*-analyses [20].

The SCCS models were fitted with the R package SCCS [21], and the rest of the analyses were conducted using SAS version 9.4 (SAS Institute Inc., Cary, North Carolina).

## 3. Results

### 3.1. Characteristics of deaths

Between December 14, 2020, and August 11, 2021, there were 9,019 non-COVID-19 deaths among individuals who received BNT162b2. Of these deaths, 69.9 % were among individuals aged 75 years or older, 50.8 % were male, and 65.5 % were non-Hispanic White (Table 1). Although the numbers of all-cause deaths and non-COVID-19 deaths were similar between males and females, more cardiac-related deaths occurred among males than females (56.9 % versus 43.1 %). Notably, there were only six cardiac-related deaths among those aged < 45 years at the six VSD sites with cause-of-death data available. These six sites accounted for 61.9 % of all-cause deaths.

Of 7,357 non-COVID-19 deaths among individuals who received mRNA-1273, 65.5 % were among individuals aged 75 years or older, 53.9 % were males, and 16.0 % were Hispanic (Table 2). The difference between the proportion of cardiac-related deaths and non-COVID-19 deaths among males was less apparent among recipients of mRNA-1273.

For Ad26.COV2.S, 55.5 % of 1,008 non-COVID-19 deaths and 54.9 % of 1,048 all-cause deaths were among those aged ≥ 75 years, and 51.3 % were females for both non-COVID-19 deaths and all-cause deaths (Table 3).

There was a total of 24,132 unvaccinated non-COVID-19 deaths, 56.6 % were among individuals aged 75 years or older, 51.0 % were males, and 17.2 % were Hispanic (Table 4).

### 3.2. Relative incidences for the primary and secondary death outcomes

The results from the SCCS models for risk intervals of 14- and 28-days after vaccination are presented in Table 5. Here, we describe point estimates of RIs and their 95 % CIs when appropriate. In instances where multiple RIs are discussed, only the point estimates and whether their 95 % CIs included 1 are mentioned due to space constraints; for the 95 % CIs of these RIs, please refer to Table 5.

For BNT162b2, after adjusting for seasonality, RI point estimates of the primary outcome (non-COVID-19 mortality) and the five secondary outcomes were below 1 across both dose 1 and dose 2 and across both risk intervals, ranging from 0.31 to 0.58, with 95 % CIs excluding 1 (Table 5).

For mRNA-1273, RI point estimates of all outcomes ranged from 0.23 to 0.80 with 95 % CIs excluding 1, except during a 14-day risk interval after the second dose, where RI point estimates were below 1 but 95 % CIs included 1 for cardiac-related mortality without pre-existing cancer and heart disease (RI = 0.78, 95 % CI, 0.58–1.04) and non-COVID-19 cardiac-related mortality without pre-existing cancer and heart disease (RI = 0.80, 95 % CI, 0.60–1.08) (Table 5). It is worth noting that RI point estimates for these two outcomes were below 1 with 95 % CIs excluding 1 after a 28-day risk interval after the second dose, with RI = 0.71 (95 % CI, 0.56–0.89) and RI = 0.73 (95 % CI, 0.58–0.91), respectively.

For Ad26.COV2.S, RIs were below 1 for non-COVID-19 mortality as well as for all-cause mortality with risk intervals of 14- and 28-days after vaccination, ranging from 0.53 to 0.67, with 95 % CIs excluding 1. For the four cardiac-related death outcomes with a risk interval of 14 days after vaccination, RIs were 0.95 (95 % CI, 0.51–1.76), 0.94 (95 % CI, 0.42–2.12), 0.98 (95 % CI, 0.53–1.82), and 0.95 (95 % CI, 0.42–2.15); and with a risk interval of 28 days after vaccination, RIs were 0.68 (95 % CI, 0.40–1.18), 0.71 (95 % CI, 0.35–1.43), 0.71 (95 % CI, 0.41–1.22), and 0.72 (95 % CI, 0.36–1.45) (Table 5).

The results for the risk interval of 10 weeks following vaccination are illustrated in Supplementary Figures 2–4. For BNT162b2, the dose 1 weekly RI point estimates for all four cardiac-related death outcomes were below 1, but 95 % CIs included 1 except that the RI point estimate for cardiac-related mortality during week 4 was slightly above 1 (RI = 1.01, 95 % CI, 0.68–1.51); some dose 2 weekly RI point estimates for all four cardiac-related death outcomes were below 1 or slightly above 1 but 95 % CIs included 1 (Supplementary Figure 2). For non-COVID-19 and all-cause deaths following dose 2, the RI increased from 0.18 to 0.74 and 0.17 to 0.74 respectively during the 10-week risk interval with 95 % CIs excluding 1.

For mRNA-1273, the dose 1 weekly RI point estimates for non-COVID-19 death and all-cause deaths were below 1 for weeks 1–4 and 7–10 with 95 % CIs excluding 1, and below 1 but 95 % CIs included 1 for weeks 5 and 6 (Supplementary Figure 3). For non-COVID-19 and all-cause deaths following dose 2, the RI increased from 0.23 to 0.83 and 0.21 to 0.83 respectively during the 10-week risk interval and 95 % CIs excluded 1.

The RI point estimates were below 1 and 95 % CI excluded 1 only for non-COVID-19 deaths one week after Ad26.COV2.S vaccination (RI = 0.31, 95 % CI, 0.22–0.44), and below 1 but 95 % CIs included 1 for the remaining weeks. The RI point estimates for all-cause deaths below 1 and 95 % CIs excluded 1 during weeks 1–4 and week 7 after Ad26.COV2. S vaccination, and below 1 but 95 % CIs included 1 for the remaining weeks (Supplementary Figure 4).

## 4. Discussion

The current study used a modified SCCS design and its results demonstrated no–increased risk of non-COVID-19 mortality and cardiac-related mortality among recipients of the three most commonly used COVID-19 vaccines in the US. Regarding cardiac-related mortality risk, our findings were consistent with those in a recent SCCS study conducted in England by Nafilyan et al. [16] and contradictory to the Florida Department of Health study. The Florida study found a statistically significant increase in cardiac-related deaths for their entire study population in the 28 days after the last dose (RI = 1.07, 95 % CI, 1.03–1.12). Our RI point estimates were lower than 1 for all four cardiac-related death outcomes among recipients of BNT162b2 and mRNA-1273 after doses 1 and 2 with 14- and 28-day risk intervals. RIs were near 1 for all four cardiac-related death outcomes among recipients of Ad26.COV2.S with a 14-day risk interval, and were below 1 with a 28-day risk interval, although RIs were not statistically different from 1. In the study by Nafilyan et al. [16] the overall RI for cardiac-related deaths was 0.84 (95 % CI, 0.61–1.15) 12 weeks after vaccination with any dose of an mRNA COVID-19 vaccine.

Compared to the Florida Department of Health study [15], the current study has several strengths. First, we accounted for the multi-dose nature of mRNA COVID-19 vaccines. In contrast, the Florida study started the observation period from the last dose and failed to consider the time between dose 1 and dose 2. By incorporating the person-time between doses, we addressed the fact that the mortality rate is zero during this interval. This inclusion of comparator person-time between doses rectifies the overestimation of risk that would occur otherwise. It was shown that disregarding the multi-dose nature results in an overestimation of risk, even in situations where there is no increased risk hypothetically [22]. Second, we included unvaccinated deaths to adjust for potential temporal effects by including month in the SCCS analyses, while the Florida study only included those who were vaccinated and died during observation period; therefore, their seasonality adjustment was not sufficient. Third, we used both cause-of-death and diagnosis/laboratory test to identify COVID-19 related deaths while the Florida study solely relied on cause-of-death data. Some cardiac-related deaths in the Florida study might have been attributable to COVID-19 disease but were not properly identified as such.

Despite including deaths in unvaccinated individuals to control for temporal trends and utilizing a modified SCCS design, residual confounding bias likely remained in our study. The bias is likely due to unmeasured time-varying confounders that may attenuate likelihood for receipt of preventive care (e.g., vaccination) as individuals approach death. The SCCS method adjusts for aspects of health seeking behavior that are time-invariant during the study period, but not for those that may vary among individuals nearing death [23]. The study has several additional limitations. First, cause-of-death data were not available in two of the VSD sites and only six VSD sites contributed data to the analyses of cardiac-related mortality. Due to limited sample size, cardiac-related mortality among males under 40 years old, the population in which the increased risk was observed in the Florida Department of Public Health study, could not be assessed. Second, due to the lack of cause-of-death data in two sites, some non-COVID-19 related deaths were potentially misclassified. Third, although the VSD population represents about 3 % of the US population, the findings from this study are more generalizable to the insured population than the entire US population. Fourth, the long-term effect of COVID-19 vaccination on mortality cannot be evaluated in this study due to the limited observation time. In addition, it may be challenging to study the long-term effect of a transient exposure (i.e., vaccination), because studying the long-term effects becomes complex due to other confounding factors.

We conclude that, using a modified SCCS design adjusting for temporal trends, no–increased risk was found for non-COVID-19 mortality, all-cause mortality, and cardiac-related mortality following the administration of the COVID-19 vaccine primary series, including BNT162b2, mRNA-1273, and Ad26.COV2.S, which supports the previously established safety of these vaccines regarding mortality risk.

## Supplementary Material

upplementary material

## Figures and Tables

**Table 1:** Characteristics of deaths among recipients of BNT162b2 during the period from December 14, 2020 to August 11, 2021.

	non-COVID-19 deaths, no. (%)	all-cause deaths, no. (%)	cardiac-related deaths, no. (%)^§^	cardiac-related deaths without pre-existing cancer and heart disease, no. (%)^§^	non-COVID-19 cardiac-related deaths, no. (%)^§^	non-COVID-19 cardiac-related deaths without pre-existing cancer and heart disease, no. (%)^§^
**Overall**	9,019 (100.0)	9,367 (100.0)	988 (100.0)	659 (100.0)	968 (100.0)	646 (100.0)
**Age (in years)**						
**12–17**	11 (0.1)	11 (0.1)	0 (0.0)	0 (0.0)	0 (0.0)	0 (0.0)
**18–44**	166 (1.8)	172 (1.8)	6 (0.6)	5 (0.8)	6 (0.6)	5 (0.8)
**45–64**	806 (8.9)	841 (9.0)	80 (8.1)	70 (10.6)	78 (8.1)	68 (10.5)
**65–74**	1,729 (19.2)	1,783 (19.0)	168 (17.0)	113 (17.1)	165 (17.0)	111 (17.2)
**75+**	6,307 (69.9)	6,560 (70.0)	734 (74.3)	471 (71.5)	719 (74.3)	462 (71.5)
**Sex**						
**Female**	4,439 (49.2)	4,609 (49.2)	426 (43.1)	280 (42.5)	418 (43.2)	275 (42.6)
**Male**	4,580 (50.8)	4,758 (50.8)	562 (56.9)	379 (57.5)	550 (56.8)	371 (57.4)
**Race/ethnicity**						
**Hispanic**	1,116 (12.4)	1,194 (12.7)	113 (11.4)	68 (10.3)	109 (11.3)	65 (10.1)
**Non-Hispanic White**	5,904 (65.5)	6,105 (65.2)	667 (67.5)	455 (69.0)	655 (67.7)	446 (69.0)
**Non-Hispanic Asian**	734 (8.1)	765 (8.2)	65 (6.6)	45 (6.8)	63 (6.5)	45 (7.0)
**Non-Hispanic Black**	541 (6.0)	557 (5.9)	56 (5.7)	34 (5.2)	55 (5.7)	34 (5.3)
**Missing**	403 (4.5)	416 (4.4)	61 (6.2)	39 (5.9)	60 (6.2)	38 (5.9)
**Multiple/Other**	321 (3.6)	330 (3.5)	26 (2.6)	18 (2.7)	26 (2.7)	18 (2.8)

§ Data from six of the eight VSD sites were included in the analysis of cardiac-related deaths, as the remaining two did not have cause-of-death data for the study period.

**Table 2:** Characteristics of deaths among recipients of mRNA-1273 during the period from December 14, 2020 to August 11, 2021.

	non-COVID-19 deaths, no. (%)	all-cause deaths, no. (%)	cardiac-related deaths, no. (%)^§^	cardiac-related deaths without pre-existing cancer and heart disease, no. (%)^§^	non-COVID-19 cardiac-related deaths, no. (%)^§^	non-COVID-19 cardiac-related deaths without pre-existing cancer and heart disease, no. (%)^§^
**Overall**	7,357 (100.0)	7,585 (100.0)	1,013 (100.0)	702 (100.0)	993 (100.0)	688 (100.0)
**Age (in years)**						
**18–44**	144 (2.0)	146 (1.9)	6 (0.6)	5 (0.7)	6 (0.6)	5 (0.7)
**45–64**	801 (10.9)	835 (11.0)	109 (10.8)	90 (12.8)	107 (10.8)	89 (12.9)
**65–74**	1,594 (21.7)	1,631 (21.5)	190 (18.8)	130 (18.5)	188 (18.9)	129 (18.8)
**75+**	4,818 (65.5)	4,973 (65.6)	708 (69.9)	477 (67.9)	692 (69.7)	465 (67.6)
**Sex**						
**Female**	3,389 (46.1)	3,498 (46.1)	459 (45.3)	309 (44.0)	445 (44.8)	299 (43.5)
**Male**	3,967 (53.9)	4,086 (53.9)	554 (54.7)	393 (56.0)	548 (55.2)	389 (56.5)
**Unknown/missing**	1 (0.0)	1 (0.0)	0 (0.0)	0 (0.0)	0 (0.0)	0 (0.0)
**Race/ethnicity**						
**Hispanic**	1,179 (16.0)	1,239 (16.3)	158 (15.6)	101 (14.4)	151 (15.2)	98 (14.2)
**Non-Hispanic White**	4,387 (59.6)	4,497 (59.3)	607 (59.9)	431 (61.4)	597 (60.1)	423 (61.5)
**Non-Hispanic Asian**	574 (7.8)	587 (7.7)	60 (5.9)	43 (6.1)	60 (6.0)	43 (6.3)
**Non-Hispanic Black**	584 (7.9)	603 (7.9)	97 (9.6)	68 (9.7)	96 (9.7)	67 (9.7)
**Missing**	365 (5.0)	387 (5.1)	56 (5.5)	38 (5.4)	54 (5.4)	36 (5.2)
**Multiple/Other**	268 (3.6)	272 (3.6)	35 (3.5)	21 (3.0)	35 (3.5)	21 (3.1)

§ Data from six of the eight VSD sites were included in the analysis of cardiac-related deaths, as the remaining two did not have cause-of-death data for the study period.

**Table 3:** Characteristics of deaths among recipients of Ad26.COV2.S during the period from February 27, 2021 to August 11, 2021.

	non-COVID-19 deaths, no. (%)	all-cause deaths, no. (%)	cardiac-related deaths, no. (%)^§^	cardiac-related deaths without pre-existing cancer and heart disease, no. (%)^§^	non-COVID-19 cardiac-related deaths, no. (%)^§^	non-COVID-19 cardiac-related deaths without pre-existing cancer and heart disease, no. (%)^§^
**Overall**	1,008 (100.0)	1,048 (100.0)	79 (100.0)	49 (100.0)	78 (100.0)	49 (100.0)
**Age (in years)**						
**18–44**	36 (3.6)	37 (3.5)	1 (1.3)	0 (0.0)	1 (1.3)	0 (0.0)
**45–64**	201 (19.9)	214 (20.4)	19 (24.1)	10 (20.4)	19 (24.4)	10 (20.4)
**65–74**	212 (21.0)	222 (21.2)	20 (25.3)	13 (26.5)	20 (25.6)	13 (26.5)
**75+**	559 (55.5)	575 (54.9)	39 (49.4)	26 (53.1)	38 (48.7)	26 (53.1)
**Sex**						
**Female**	517 (51.3)	538 (51.3)	32 (40.5)	20 (40.8)	32 (41.0)	20 (40.8)
**Male**	491 (48.7)	510 (48.7)	47 (59.5)	29 (59.2)	46 (59.0)	29 (59.2)
**Race/ethnicity**						
**Hispanic**	153 (15.2)	164 (15.6)	12 (15.2)	7 (14.3)	12 (15.4)	7 (14.3)
**Non-Hispanic White**	602 (59.7)	615 (58.7)	45 (57.0)	25 (51.0)	44 (56.4)	25 (51.0)
**Non-Hispanic Asian**	77 (7.6)	80 (7.6)	5 (6.3)	4 (8.2)	5 (6.4)	4 (8.2)
**Non-Hispanic Black**	104 (10.3)	114 (10.9)	10 (12.7)	8 (16.3)	10 (12.8)	8 (16.3)
**Missing**	32 (3.2)	33 (3.1)	4 (5.1)	4 (8.2)	4 (5.1)	4 (8.2)
**Multiple/Other**	40 (4.0)	42 (4.0)	3 (3.8)	1 (2.0)	3 (3.8)	1 (2.0)

§ Data from six of the eight VSD sites were included in the analysis of cardiac-related deaths, as the remaining two did not have cause-of-death data for the study period.

**Table 4:** Characteristics of deaths among unvaccinated individuals during the period from December 14, 2020 to August 11, 2021.

	non-COVID-19 deaths, no. (%)	all-cause deaths, no. (%)	cardiac-related deaths, no. (%)^§^	cardiac-related deaths without pre-existing cancer and heart disease, no. (%)^§^	non-COVID-19 cardiac-related deaths, no. (%)^§^	non-COVID-19 cardiac-related deaths without pre-existing cancer and heart disease, no. (%)^§^
**Overall**	24,132 (100.0)	31,666 (100.0)	3,062 (100.0)	1,883 (100.0)	2,835 (100.0)	1,757 (100.0)
**Age (in years)**						
**12–17**	61 (0.3)	65 (0.2)	3 (0.1)	2 (0.1)	2 (0.1)	1 (0.1)
**18–44**	1,247 (5.2)	1,513 (4.8)	73 (2.4)	62 (3.3)	68 (2.4)	58 (3.3)
**45–64**	4,280 (17.7)	5,972 (18.9)	425 (13.9)	324 (17.2)	405 (14.3)	312 (17.8)
**65–74**	4,895 (20.3)	6,771 (21.4)	555 (18.1)	359 (19.1)	516 (18.2)	336 (19.1)
**75+**	13,649 (56.6)	17,345 (54.8)	2,006 (65.5)	1,136 (60.3)	1,844 (65.0)	1,050 (59.8)
**Sex**						
**Female**	11,826 (49.0)	14,805 (46.8)	1,333 (43.5)	798 (42.4)	1,245 (43.9)	748 (42.6)
**Male**	12,304 (51.0)	16,858 (53.2)	1,728 (56.4)	1,084 (57.6)	1,589 (56.1)	1,008 (57.4)
**Unknown/missing**	2 (0.0)	3 (0.0)	1 (0.0)	1 (0.0)	1 (0.0)	1 (0.0)
**Race/ethnicity**						
**Hispanic**	4,152 (17.2)	7,141 (22.6)	487 (15.9)	317 (16.8)	426 (15.0)	278 (15.8)
**Non-Hispanic White**	13,526 (56.1)	16,138 (51.0)	1,782 (58.2)	1,065 (56.6)	1,673 (59.0)	1,010 (57.5)
**Non-Hispanic Asian**	1,824 (7.6)	2,525 (8.0)	181 (5.9)	112 (5.9)	159 (5.6)	101 (5.7)
**Non-Hispanic Black**	2,263 (9.4)	2,874 (9.1)	333 (10.9)	196 (10.4)	316 (11.1)	185 (10.5)
**Missing**	1,402 (5.8)	1,707 (5.4)	191 (6.2)	129 (6.9)	178 (6.3)	122 (6.9)
**Multiple/Other**	965 (4.0)	1,281 (4.0)	88 (2.9)	64 (3.4)	83 (2.9)	61 (3.5)

§ Data from six of the eight VSD sites were included in the analysis of cardiac-related deaths, as the remaining two did not have cause-of-death data for the study period.

**Table 5:** Relative incidences of non-COVID-19 mortality, all-cause mortality, cardiac-related mortality, cardiac-related mortality without pre-existing cancer and heart disease, non-COVID-19 cardiac-related mortality, and non-COVID-19 cardiac-related mortality without pre-existing cancer and heart disease with 14- and 28-day risk intervals following COVID-19 vaccination during the period from December 14, 2020 to August 11, 2021.

		Relative incidences (95 % confidence interval) 14-day risk interval		28-day risk interval	
Vaccines	Outcomes	Dose 1	Dose 2	Dose 1	Dose 2
BNT162b2	non-COVID-19 mortality	0.34 (0.31–0.38)	0.39 (0.35–0.43)	0.44 (0.41–0.47)	0.46 (0.43–0.50)
	all-cause mortality	0.31 (0.28–0.34)	0.36 (0.32–0.40)	0.41 (0.38–0.44)	0.44 (0.41–0.47)
	cardiac-related mortality^§^	0.43 (0.32–0.57)	0.54 (0.41–0.72)	0.45 (0.37–0.56)	0.53 (0.43–0.65)
	cardiac-related mortality without pre-existing cancer and heart disease^§^	0.52 (0.37–0.72)	0.58 (0.41–0.81)	0.47 (0.37–0.61)	0.52 (0.40–0.67)
	non-COVID-19 cardiac-related mortality^§^	0.43 (0.32–0.58)	0.57 (0.43–0.76)	0.45 (0.36–0.56)	0.55 (0.44–0.67)
	non-COVID-19 cardiac-related mortality without pre-existing cancer and heart disease^§^	0.51 (0.36–0.72)	0.60 (0.43–0.85)	0.46 (0.35–0.60)	0.54 (0.42–0.70)
mRNA-1273	non-COVID-19 mortality	0.26 (0.23–0.29)	0.41 (0.37–0.46)	0.31 (0.29–0.34)	0.48 (0.45–0.52)
	all-cause mortality	0.23 (0.20–0.26)	0.39 (0.35–0.44)	0.29 (0.27–0.31)	0.46 (0.43–0.50)
	cardiac-related mortality^§^	0.26 (0.18–0.36)	0.67 (0.52–0.86)	0.40 (0.33–0.49)	0.69 (0.57–0.83)
	cardiac-related mortality without pre-existing cancer and heart disease^§^	0.26 (0.17–0.41)	0.78 (0.58–1.04)	0.42 (0.33–0.54)	0.71 (0.56–0.89)
	non-COVID-19 cardiac-related mortality^§^	0.26 (0.18–0.38)	0.69 (0.54–0.90)	0.42 (0.34–0.52)	0.71 (0.59–0.86)
	non-COVID-19 cardiac-related mortality without pre-existing cancer and heart disease^§^	0.27 (0.17–0.42)	0.80 (0.60–1.08)	0.44 (0.34–0.57)	0.73 (0.58–0.91)
Ad26.COV2.S	non-COVID-19 mortality	0.53 (0.43–0.66)	N/A	0.66 (0.57–0.76)	N/A
	all-cause mortality	0.55 (0.45–0.67)	N/A	0.67 (0.58–0.77)	N/A
	cardiac-related mortality^§^	0.95 (0.51–1.76)	N/A	0.68 (0.40–1.18)	N/A
	cardiac-related mortality without pre-existing cancer and heart disease^§^	0.94 (0.42–2.12)	N/A	0.71 (0.35–1.43)	N/A
	non-COVID-19 cardiac-related mortality^§^	0.98 (0.53–1.82)	N/A	0.71 (0.41–1.22)	N/A
	non-COVID-19 cardiac-related mortality without pre-existing cancer and heart disease^§^	0.95 (0.42–2.15)	N/A	0.72 (0.36–1.45)	N/A

§ Data from six of the eight VSD sites were included in the analysis of cardiac-related deaths, as the remaining two did not have cause-of-death data for the study period. N/A, not applicable.

## Data Availability

Data will be made available on request.
