# Supplementary material for: Mortality risk after COVID-19 vaccination: A self-controlled case series study
Source: Vaccine. Author manuscript; Available in PMC 2024 Jul 11. (PMC11238073; doi:10.1016/j.vaccine.2024.02.032)
Supplement: upplementary material [file NIHMS2007681-supplement-upplementary_material.docx]

Supplementary Figure 1. Relationship among six death outcomes

excluding COVID-19 related deaths

cardiac-related deaths

without pre-existing

cancer and heart disease

cardiac-related deaths

non-COVID-19 cardiac-related deaths

non-COVID-19 cardiac-related deaths without

pre-existing cancer and heart disease

excluding deaths with pre-existing

cancer and heart disease

selecting deaths with cause-of-death being cardiac

all-cause deaths

non-COVID-19 deaths

Supplementary Figure 2. Overall and weekly relative incidences of primary and secondary outcomes 10 weeks after BNT162b2 vaccination^§^

Dose 1 Dose 2


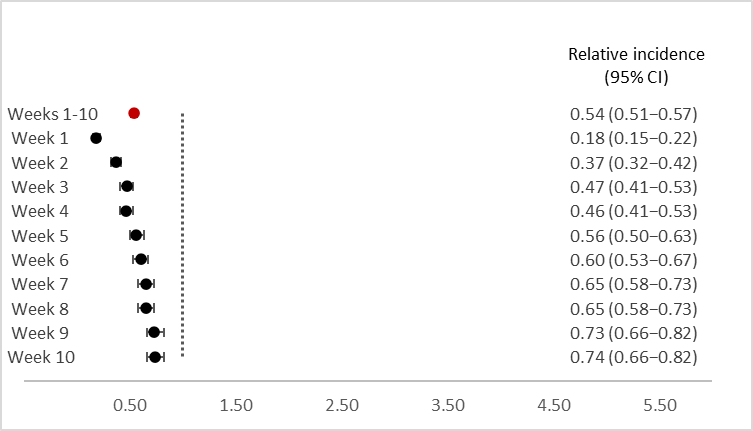

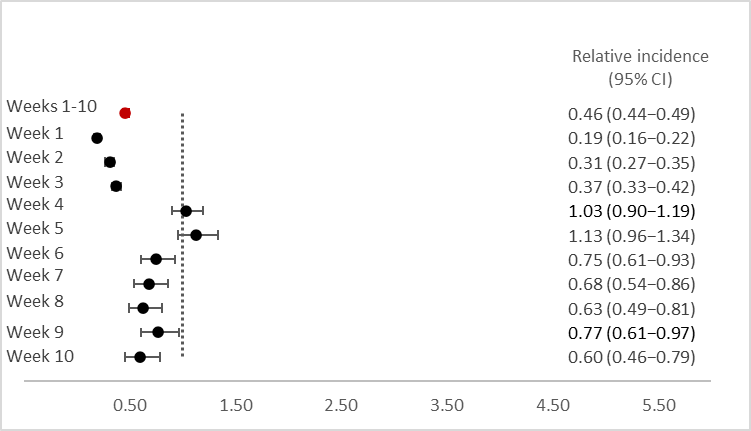


Non-COVID-19 mortality


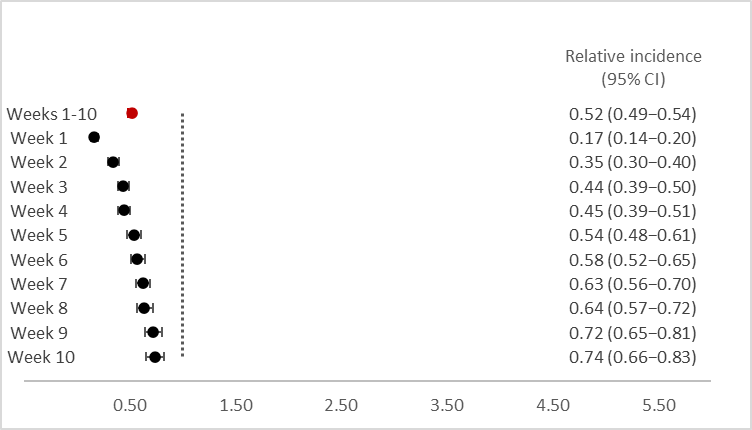

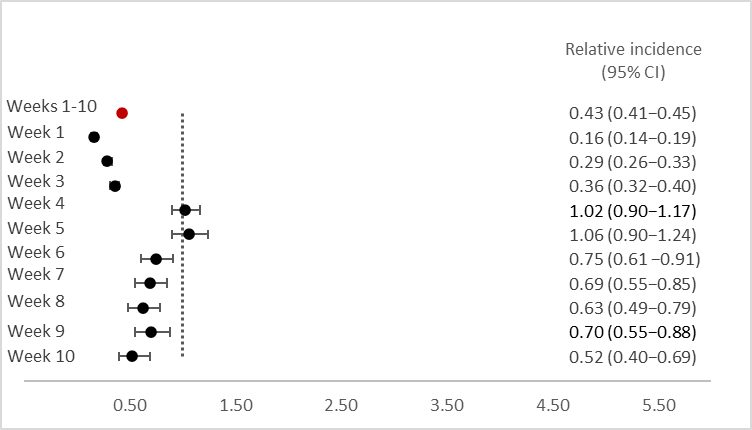


All-cause mortality


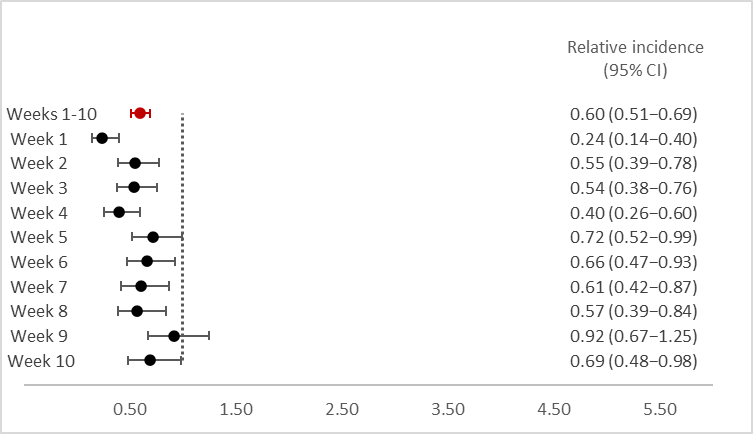

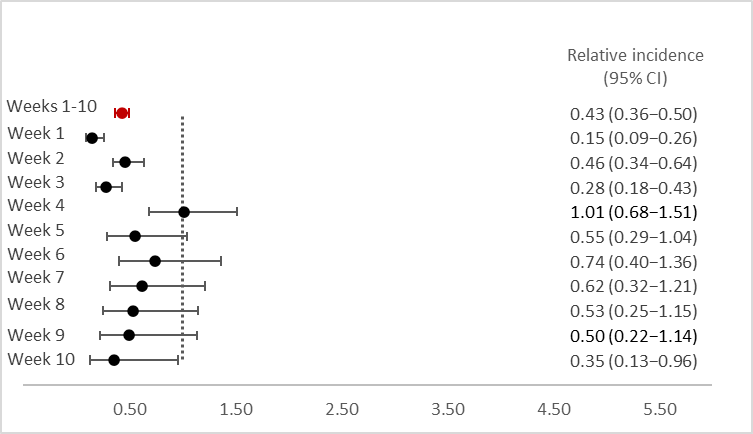


Cardiac-related mortality

Supplementary Figure 2 (continued).


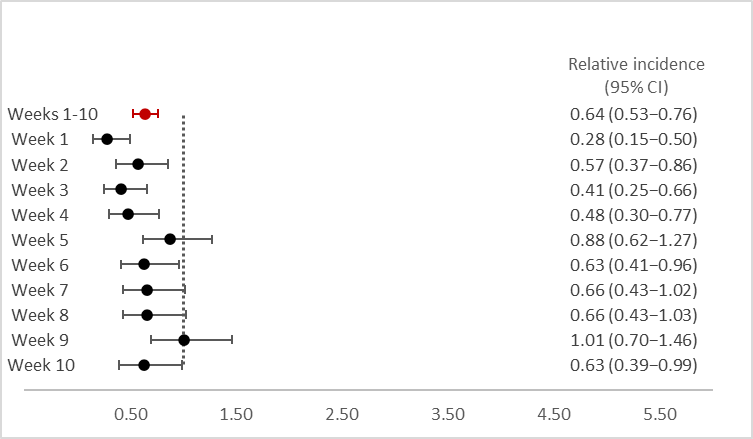

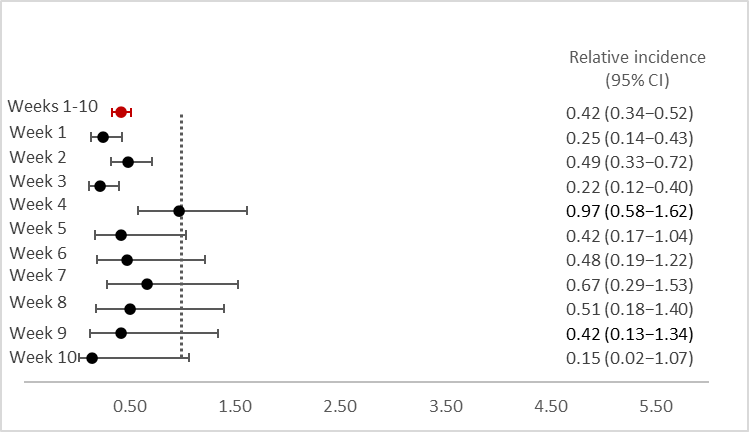


Cardiac-related mortality

excluding pre-existing cancer and heart disease


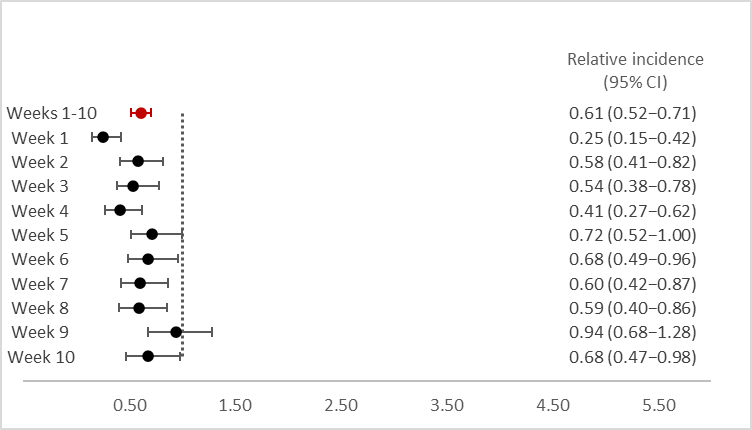

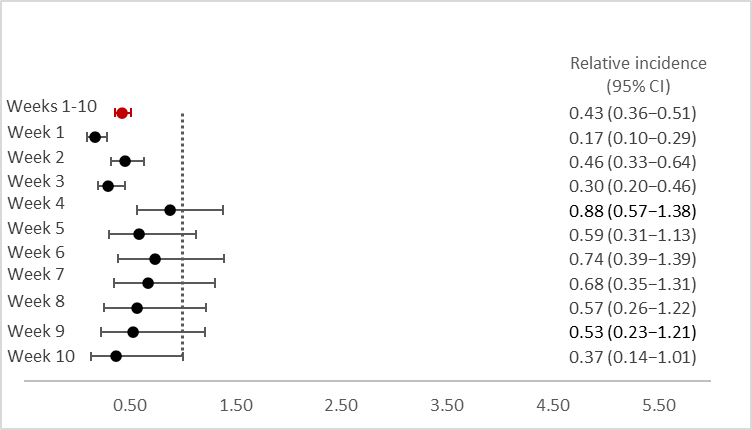


Non-COVID-19 cardiac-related mortality


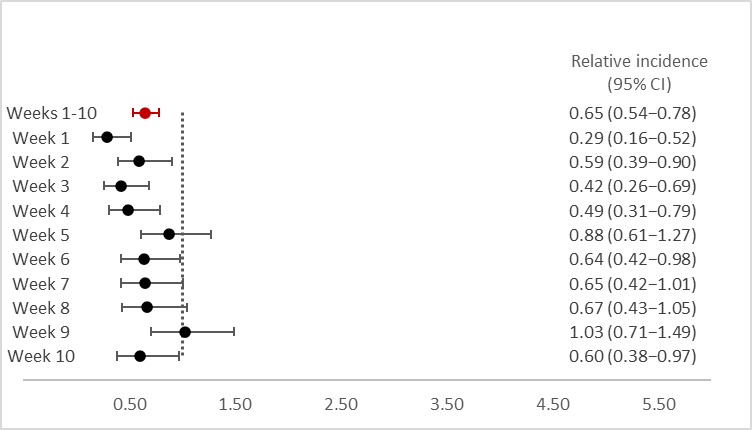

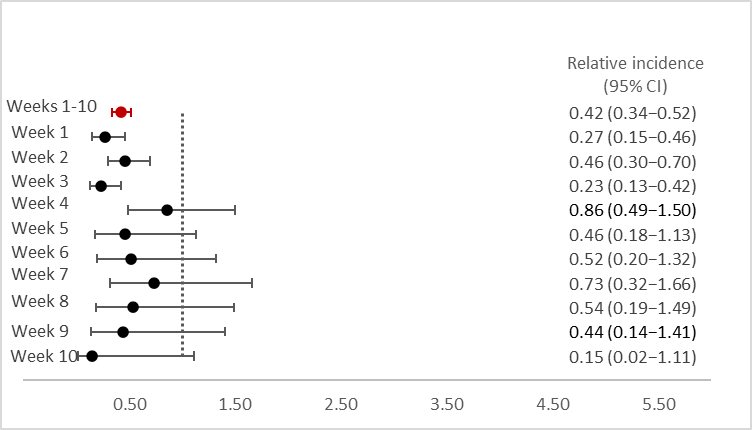


Non-COVID-19 cardiac-related mortality excluding pre-existing cancer and heart disease

^§^Data from six of the eight VSD sites were included in the analysis of cardiac-related deaths, as the remaining two did not have cause-of-death data for the study period. CI, confidence interval.

Supplementary Figure 3. Overall and weekly relative incidences of primary and secondary outcomes 10 weeks after mRNA-1273 vaccination^§^

Dose 1 Dose 2


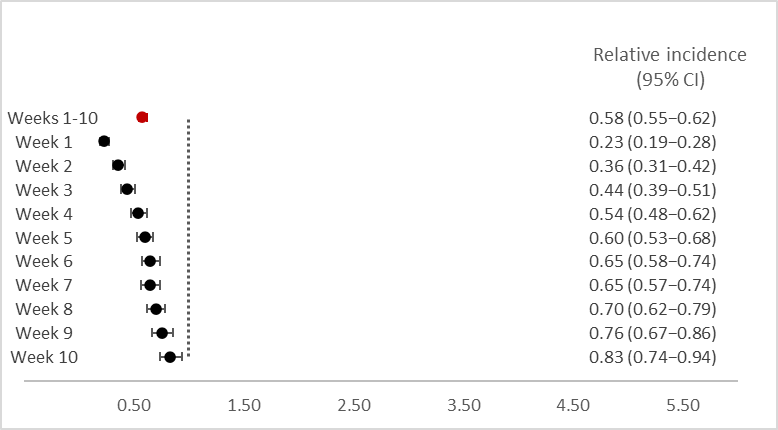

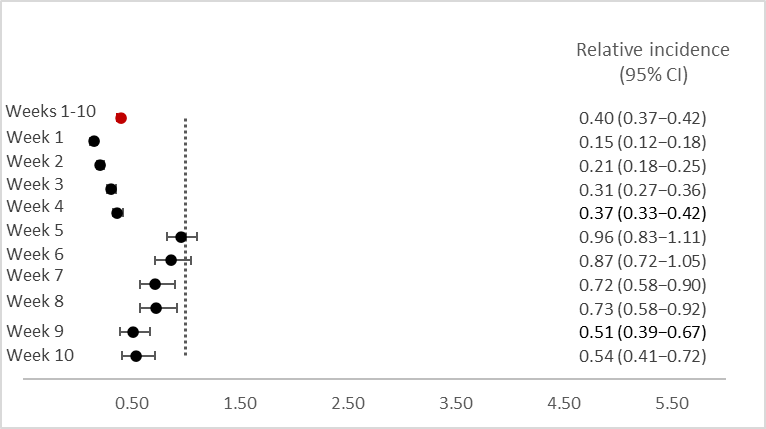


Non-COVID-19 mortality


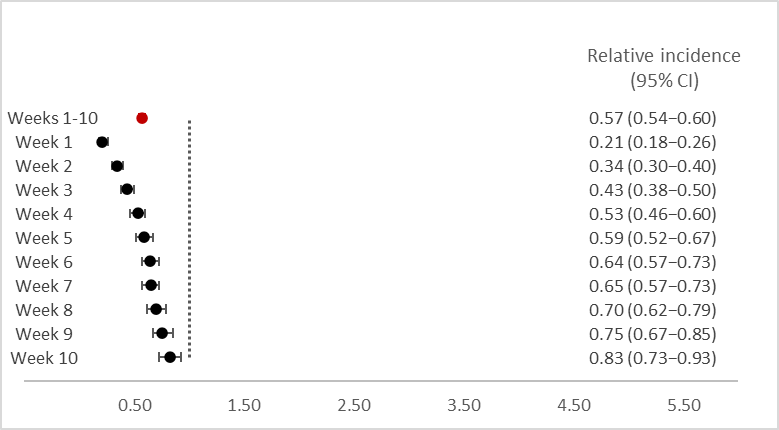

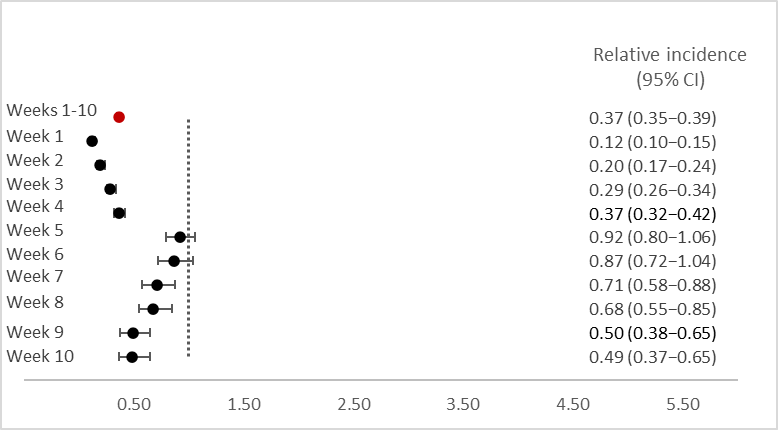


All-cause mortality


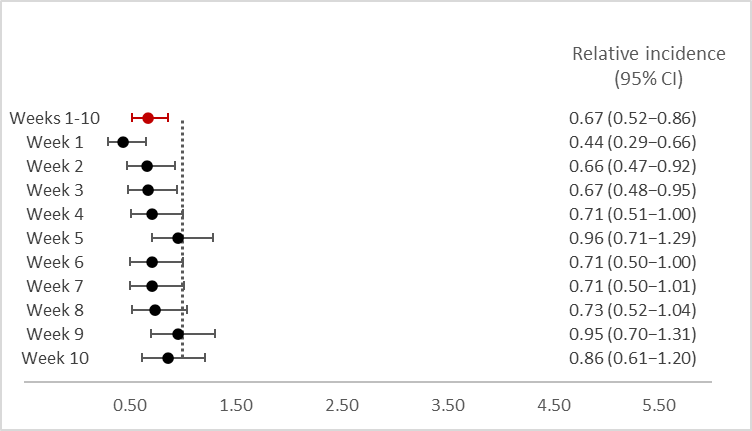

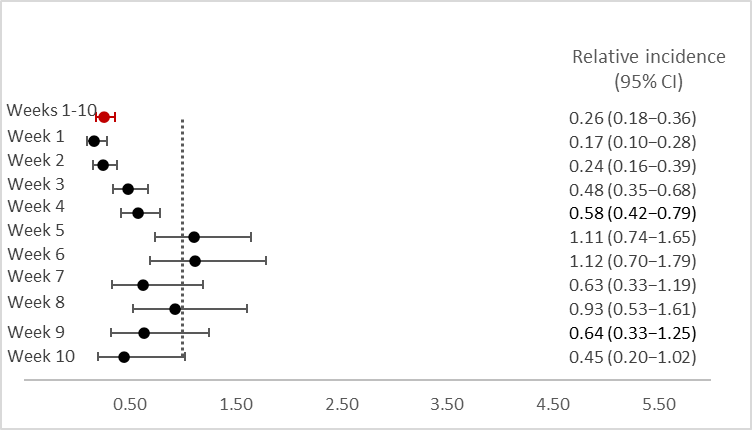


Cardiac-related mortality


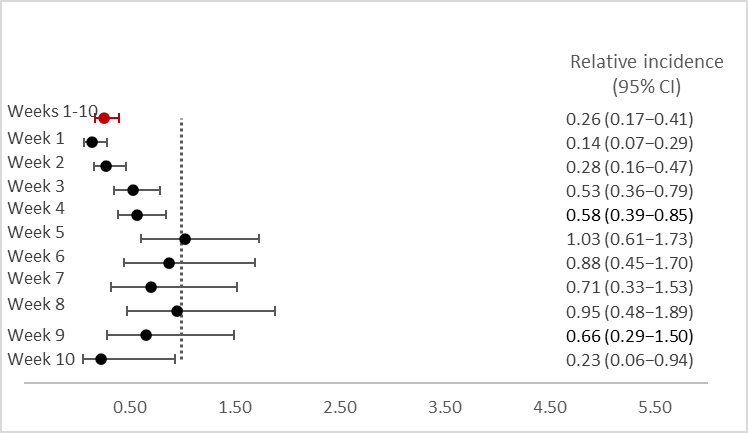

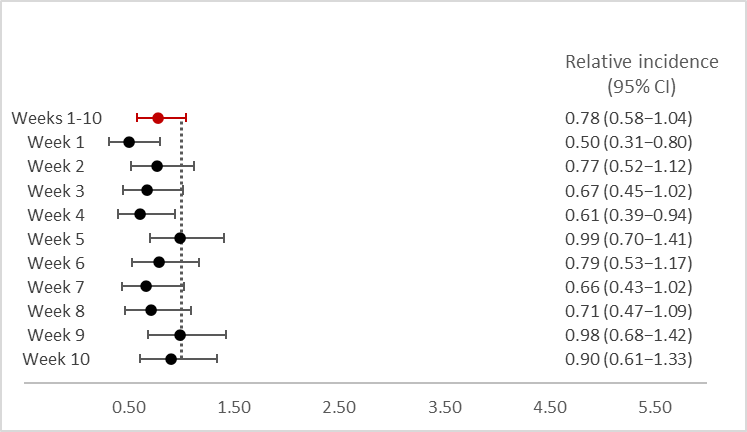
Supplementary Figure 3 (continued).

Cardiac-related mortality

excluding pre-existing cancer and heart disease


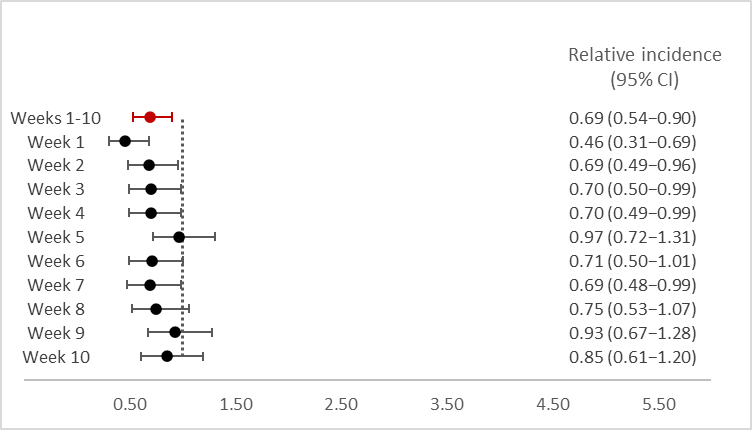

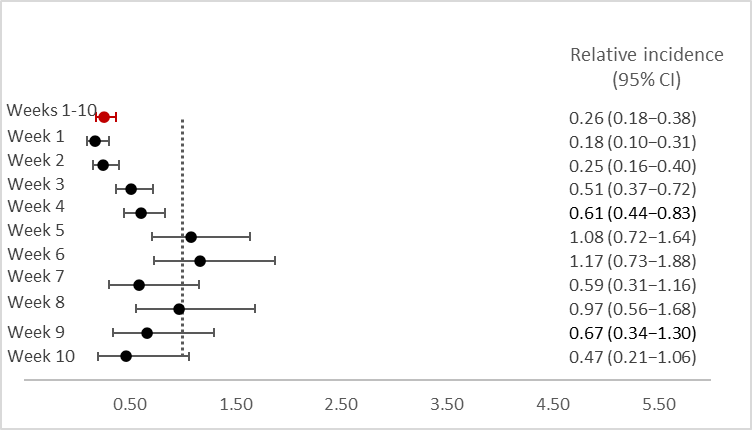


Non-COVID-19 cardiac-related mortality


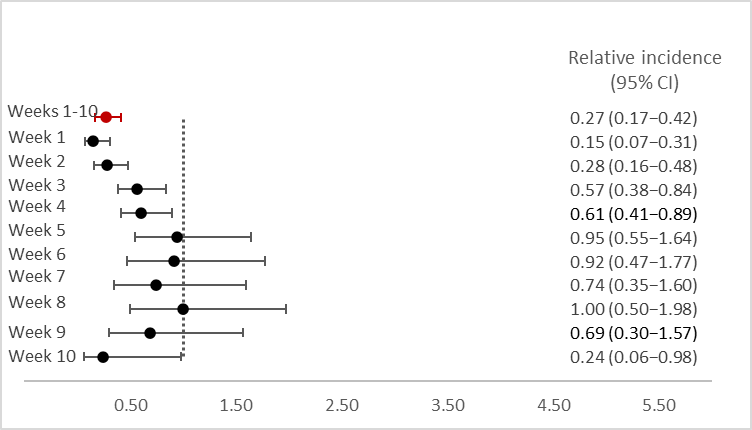

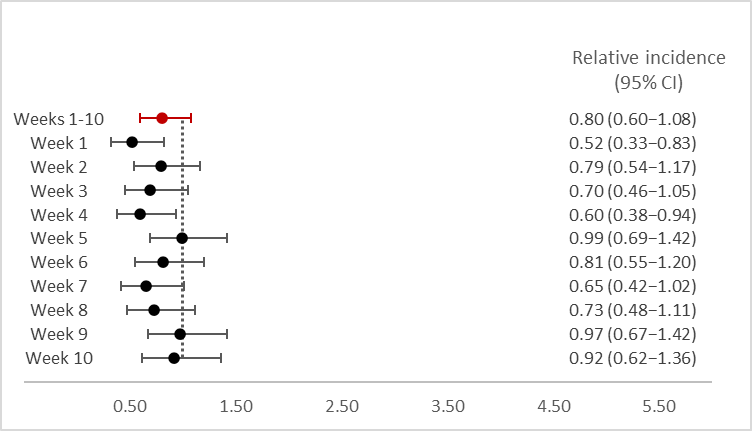


Non-COVID-19 cardiac-related mortality excluding pre-existing cancer and heart disease

^§^Data from six of the eight VSD sites were included in the analysis of cardiac-related deaths, as the remaining two did not have cause-of-death data for the study period. CI, confidence interval.

Supplementary Figure 4. Overall and weekly relative incidences of primary and secondary outcomes 10 weeks after Ad26.COV2.S vaccination^§^

^
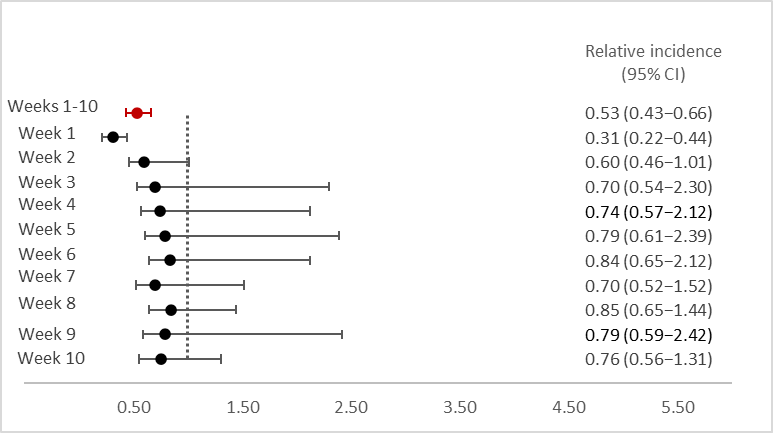
^
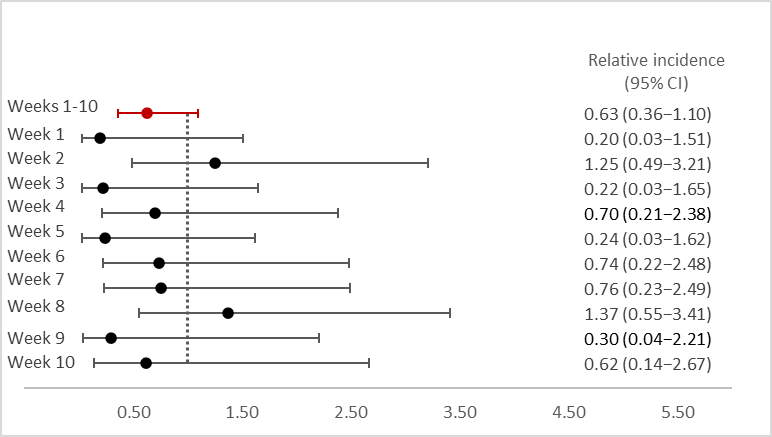


Non-COVID-19 mortality

Cardiac-related mortality

excluding pre-existing cancer and heart disease


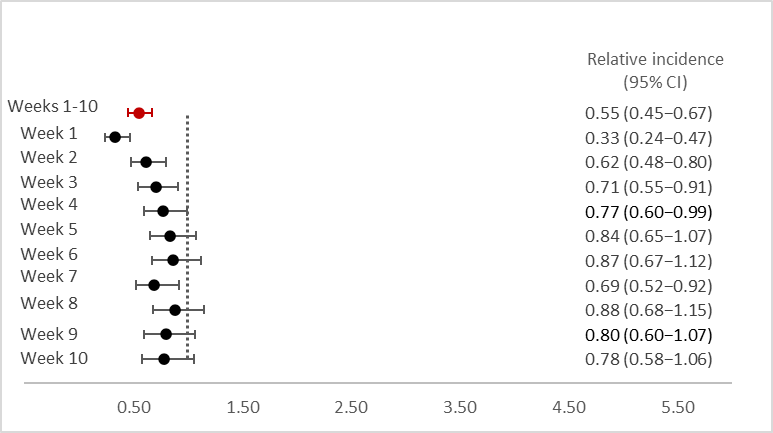

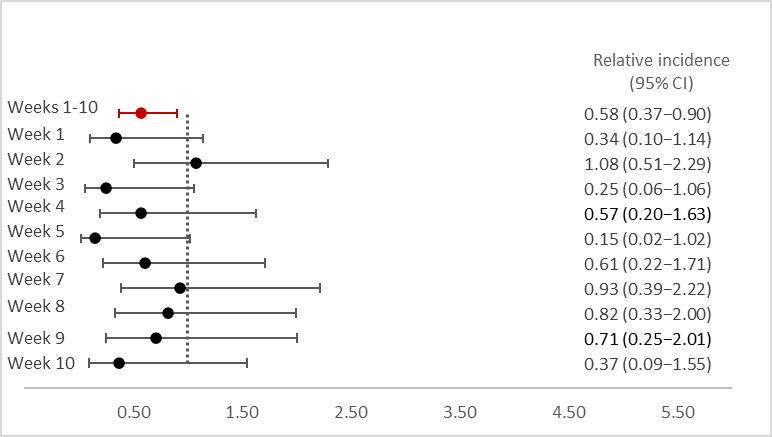


All-cause mortality

Non-COVID-19 cardiac-related mortality


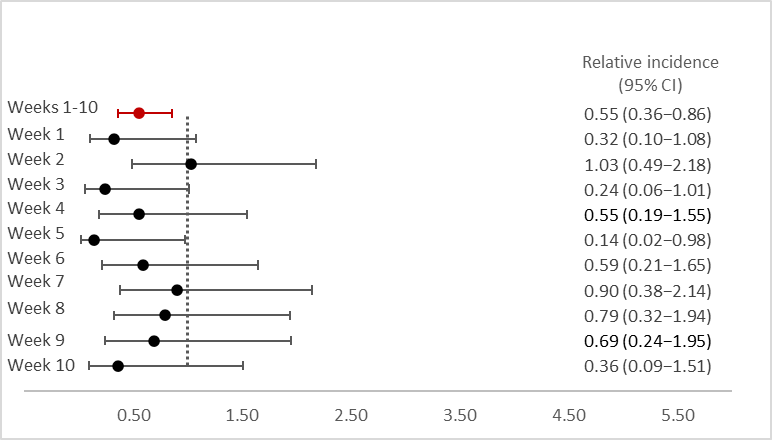

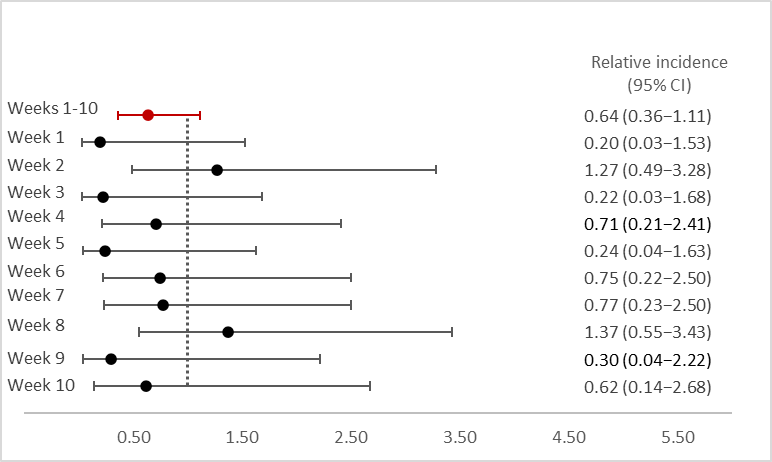


Cardiac-related mortality

Non-COVID-19 cardiac-related mortality excluding pre-existing cancer and heart disease

^§^Data from six of the eight VSD sites were included in the analysis of cardiac-related deaths, as the remaining two did not have cause-of-death data for the study period. CI, confidence interval.
